# Blood-brain barrier breakdown in COVID-19 ICU survivors: an MRI pilot study

**DOI:** 10.1515/nipt-2023-0018

**Published:** 2023-11-22

**Authors:** Wen Shi, Dengrong Jiang, Hannah Rando, Shivalika Khanduja, Zixuan Lin, Kaisha Hazel, George Pottanat, Ebony Jones, Cuimei Xu, Doris Lin, Sevil Yasar, Sung-Min Cho, Hanzhang Lu

**Affiliations:** Department of Biomedical Engineering, Johns Hopkins University School of Medicine, Baltimore, MD, USA; The Russell H. Morgan Department of Radiology & Radiological Science, Johns Hopkins University School of Medicine, Baltimore, MD, USA; Department of Surgery, Johns Hopkins University School of Medicine, Baltimore, MD, USA; Department of Medicine, Johns Hopkins University School of Medicine, Baltimore, MD, USA; Department of Neurology, Neurosurgery, Surgery, Anesthesiology, and Critical Care Medicine, Johns Hopkins University School of Medicine, Baltimore, MD, USA; F. M. Kirby Research Center for Functional Brain Imaging, Kennedy Krieger Research Institute, Baltimore, MD, USA

**Keywords:** blood-brain barrier, COVID-19, MRI, permeability, WEPCAST

## Abstract

**Objectives:**

Coronavirus disease 2019 (COVID-19) results in severe inflammation at the acute stage. Chronic neuroinflammation and abnormal immunological response have been suggested to be the contributors to neuro-long-COVID, but direct evidence has been scarce. This study aims to determine the integrity of the blood-brain barrier (BBB) in COVID-19 intensive care unit (ICU) survivors using a novel MRI technique.

**Methods:**

COVID-19 ICU survivors (n=7) and age and sex-matched control participants (n=17) were recruited from June 2021 to March 2023. None of the control participants were hospitalized due to COVID-19 infection. The COVID-19 ICU survivors were studied at 98.6 ± 14.9 days after their discharge from ICU. A non-invasive MRI technique was used to assess the BBB permeability to water molecules, in terms of permeability surface area-product (PS) in the units of mL/100 g/min.

**Results:**

PS was significantly higher in COVID-19 ICU survivors (p=0.038) when compared to the controls, with values of 153.1 ± 20.9 mL/100 g/min and 132.5 ± 20.7 mL/100 g/min, respectively. In contrast, there were no significant differences in whole-brain cerebral blood flow (p=0.649) or brain volume (p=0.471) between the groups.

**Conclusions:**

There is preliminary evidence of a chronic BBB breakdown in COVID-19 survivors who had a severe acute infection, suggesting a plausible contributor to neurological long-COVID symptoms.

## Introduction

Coronavirus disease 2019 (COVID-19) induced by severe acute respiratory syndrome coronavirus (SARS-CoV-2) has caused an unprecedented health crisis [1]. Despite the present successful control of COVID-19 in the acute phase, the long-term sequelae of COVID-19, including those on the brain and neuropsychiatric, remain a major medical burden [2]. Prior studies reported that severe COVID-19 infection resulted in prevalent neurologic symptoms and long-term neurocognitive deficits including brain fog [3, 4], cognitive decline [5], and psychiatric symptoms [6]. However, the underlying pathophysiological mechanisms of these symptoms are still poorly understood [7].

Inflammation is thought to be one major contributor to brain abnormalities in severe COVID-19 [8, 9]. Prior studies have revealed that increased pro-inflammatory cytokines such as interleukin-10 (IL-10), granulocyte colony-stimulating factor (GCSF), and tumor necrosis factor (TNFα) were observed in intensive care unit (ICU) patients compared with non-ICU patients [10]. Serum cytokines induced by systemic inflammation can impact blood-brain barrier (BBB) function and promote BBB disruption. At a chronic phase, it has also been reported that proteins related to neuroinflammation such as matrix metalloproteinase 9 (MMP-9) and glial fibrillary acidic protein (GFAP) were elevated in patients with neurological symptoms [11]. It is therefore plausible that BBB breakdown is a long-lasting pathophysiological alteration that will persist beyond the acute phase of COVID-19. However, to date, BBB assessment in COVID-19 patients has been limited, part of which is due to the scarcity of tools to probe BBB function in humans [12, 13].

A novel magnetic resonance imaging (MRI) technique, referred to as water-extraction-with-phase-contrast-arterial-spin-tagging (WEPCAST) MRI, was developed to non-invasively measure BBB permeability to water, without requiring exogenous (gadolinium) contrast agents [14, 15]. The goal of the present study was therefore to use WEPCAST MRI to assess BBB integrity in COVID-19 patients at a chronic phase. We note that patients who underwent ICU hospitalization are most likely to have experienced severe cytokine storm thereby suffering from long-lasting BBB damage. Therefore, in this pilot study, we recruited a group of COVID-19 ICU survivors approximately 3 months after their discharge from the ICU and compared them to a group of age and sex-matched control participants without any history of hospitalization due to COVID-19.

## Results

The demographic characteristics of COVID-19 ICU survivors and age and sex-matched healthy controls are shown in Table 1. For COVID-19 ICU survivors, the average time from symptom onset to ICU admission was 2.9 ± 3.2 days, with a duration of 20.0 ± 18.2 days in ICU after infection. Two of the patients were intubated and four of them underwent ventilator support during hospitalization.

**Table 1:** Demographic information of COVID-19 ICU survivors and controls (mean ± SD).

Characteristic	COVID-19 ICU survivors (n=7)	Controls (n=17)	*p*-Value
Age, years	47.0 ± 23.5	46.7 ± 22.5	0.900^a^
Female, n, %	4 (57 %)	11 (65 %)	0.728^b^
Time from symptom onset to ICU admission, days	2.9 ± 3.2	–	–
Intubation, n, %	2 (29 %)	–	–
Ventilator support, n, %	4 (57 %)	–	–
Duration in ICU, days	20.0 ± 18.2	–	–
Time from ICU discharge to MRI examination, days	98.6 ± 14.9	–	–

^a^Wilcoxon signed-rank test, ^b^Chi-square test.

**Figure 1:**
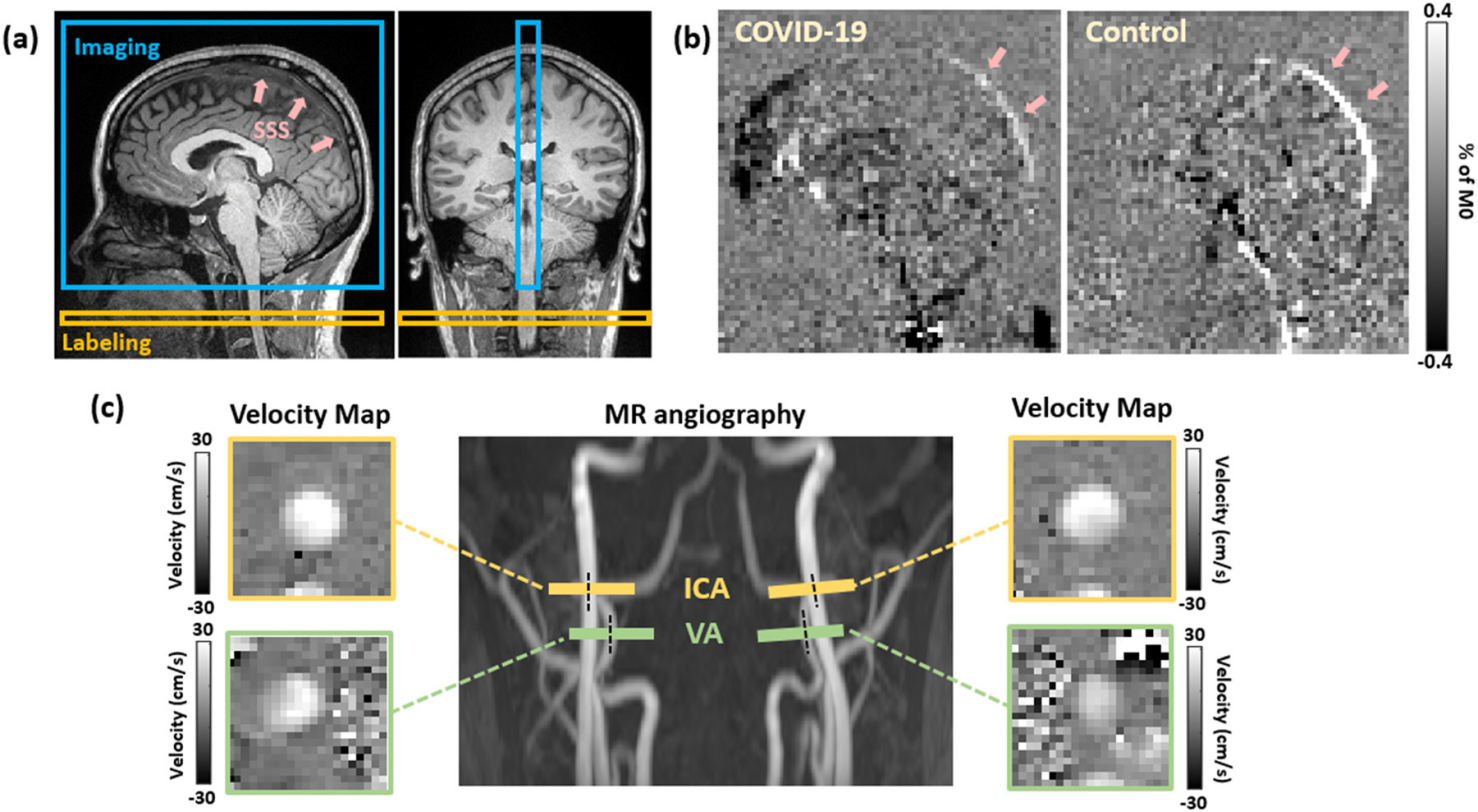
MRI technique used for the assessment of BBB permeability to water. (a) Typical imaging (blue box) and labeling position (orange box) of WEPCAST MRI. SSS, superior sagittal sinus. (b) Representative WEPCAST images of a COVID-19 ICU survivor (72 years, M) and a control subject (72 years, F). (c) Typical imaging positions for the phase contrast (PC) MRI based on the maximum intensity projection of the MR angiography. Global cerebral blood flow (CBF) was measured from the four feeding arteries, i.e., left/right internal carotid arteries (ICA) and left/right vertebral arteries (VA). The corresponding velocity maps of the four arteries are shown at the corners. **Participants**: 7 COVID-19 ICU survivors (47.0 ± 23.5 years, 3 M/4F, duration of ICU stay=20.0 ± 18.2 days) and 17 age- and sex-matched healthy controls (46.7 ± 22.5 years, 6M/11F) were recruited from June 2021 to March 2023. The COVID-19 ICU survivors were recruited by an intensive care physician (S.C.). The controls were recruited using flyers placed on campus and in the community. Ten of the control participants were scanned between June 2021 and August 2021, and they most likely have never had COVID-19 infection. The other seven of the controls were scanned between April 2022 and March 2023, and some of them may have had COVID-19 infection. None of the control participants have been hospitalized due to COVID-19 infection. All participants provided written informed consent approved by Johns Hopkins University Institutional Review Board. The COVID-19 survivors were scanned at approximately 3 months (98.6 ± 14.9 days) after their discharge from ICU. **Experiment:** all MRI scans were conducted on a Siemens 3T Prisma scanner (Siemens Healthineers, Erlangen, Germany) with a 32-channel head coil. The WEPCAST MRI technique measures BBB permeability to water by labeling the water molecules in the incoming arteries and, by determining the fraction of the water that remained in the vessel versus those exchanged into the brain tissue at the capillary-tissue interface, one can obtain an estimation of the water extraction fraction (*E*). The measurement is performed in the major draining vein of the brain, typically the superior sagittal sinus (SSS), yielding a whole-brain measure of *E*. Additionally, global CBF, f, is obtained by PC velocity MRI and normalized by whole-brain volume as measured by a 1 mm^3^ isotropic-resolution T1-MPRAGE scan. Finally, the BBB permeability index, referred to as permeability-surface-area product (PS), was calculated from *E* and f according to the Renkin–Crone model: PS=−*ln*(1 − *E*)·f. More technical details can be found somewhere else [14, 15]. **Imaging protocol**: WEPCAST MRI was performed with the following parameters: TR/TE=9200/9.5 ms, FOV=200 × 200 mm^2^, voxel size=3.1 × 3.1 × 10 mm^3^, labeling duration=4000 ms, post-labeling delay=3000 ms, VENC=20 cm/s, GRAPPA=3. PC velocity MRI was performed with the following two protocols: TR=13 (16) ms, TE=8 (10) ms, FOV=200 × 200 mm^2^, voxel size=0.5 × 0.5 × 5 mm^3^, flip angle=15°, VENC=60 (40) cm/s.

Figure 1a shows the typical mid-sagittal imaging plane and labeling position of WEPCAST MRI. Figure 1b shows representative WEPCAST images in a COVID-19 ICU survivor (72 years, M) and a control participant (72 years, F). We also measured whole-brain cerebral blood flow (CBF) using phase-contrast (PC) MRI. Figure 1c shows the position of the PC scans based on the MR angiography, as well as representative velocity maps in internal carotid and vertebral arteries.

**Figure 2:**
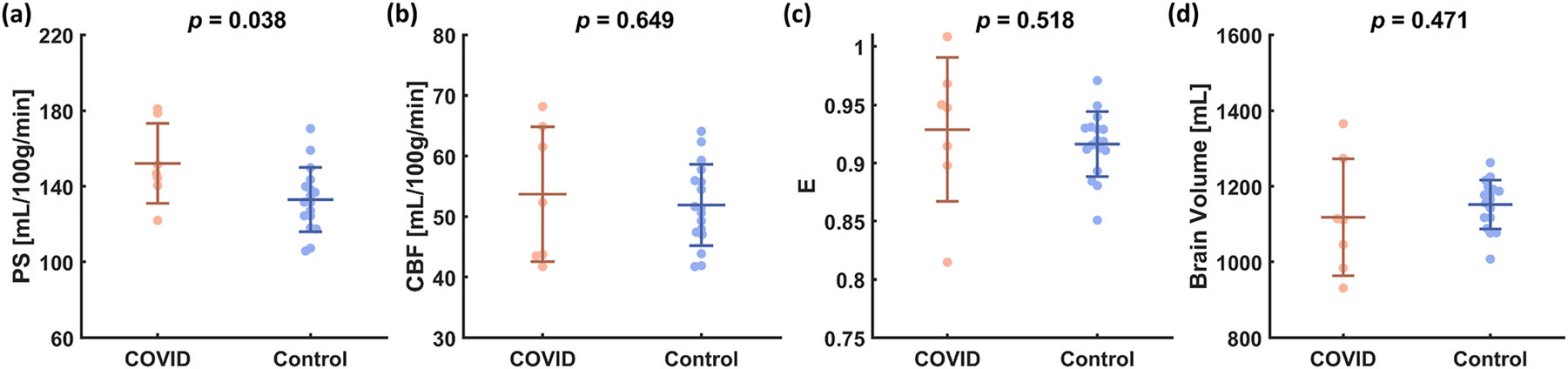
Comparison of (a) PS, (b) CBF, (c) *E* and (d) brain volume between COVID-19 ICU survivors and controls. Each dot represents data from one participant. Note that all variables have been adjusted for age and sex using linear regression. PS, permeability-surface area product; CBF, cerebral blood flow; *E*, water extraction fraction. **Statistical analysis**: analysis was conducted using in-house MATLAB scripts (version R2021a, Mathworks, Natick, MA). CBF, *E*, PS values and brain volume between the COVID-19 ICU survivors and controls were compared using linear regression in which the group assignment was the independent variable, and age and sex were covariates. The association between CBF and *E* was also evaluated by linear regression. A p-value of less than 0.05 was considered statistically significant.

The results of quantitative comparisons are shown in Figure 2. All variables have been adjusted for age and sex using linear regression. Figure 2a showed that BBB permeability-area-surface product (PS) is significantly higher in COVID-19 ICU survivors when compared to controls (p=0.038). The average values were 153.1 ± 20.9 mL/100 g/min for the COVID-19 ICU survivors and 132.5 ± 20.7 mL/100 g/min for the controls. The whole-brain cerebral blood flow (p=0.649, Figure 2b) and water extraction fraction (*E*, p=0.518, Figure 2c) were not different between the two participant groups. We also evaluated the brain volume and there was not a significant difference between the groups, in terms of gray matter (p=0.444), white matter (p=0.549), or whole brain volume (p=0.471, Figure 2d).

**Figure 3:**
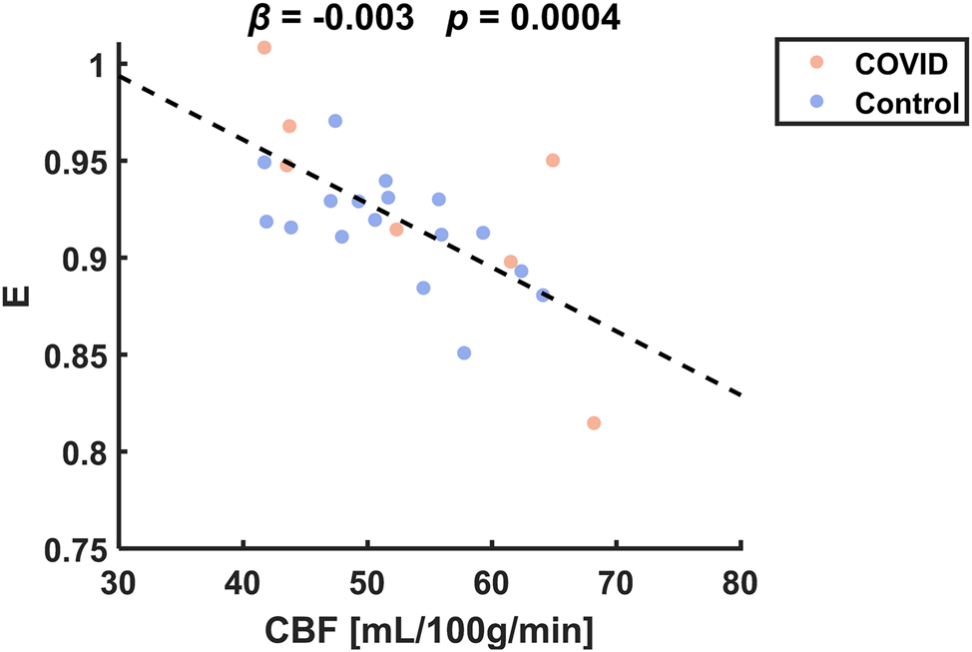
Correlation between CBF and *E* across individuals. Each dot represents data from one participant, with COVID-19 ICU survivors and controls shown in different colors. β and p-value are the linear regression results. CBF, cerebral blood flow; *E*, water extraction fraction.

Since the PS estimation was computed from CBF and *E*, we also investigated the reason that CBF and showed no group difference but PS did. We found a significant inverse relationship between CBF and *E* (Figure 3, β=−0.003, p=0.0004), suggesting that CBF and *E* are inter-related physiological parameters but their combination using the biophysical model (listed in Figure 1 legend) can cancel the physiological noise and allow more accurate assessment of the BBB property, i.e., PS.

## Discussion

In this study, we used a novel WEPCAST MRI technique to measure BBB permeability in COVID-19 ICU survivors. A significantly higher PS value was observed in the COVID-19 ICU survivors when compared to control participants, suggesting a chronic BBB breakdown in severe COVID-19 patients and long-lasting damage and inflammation of COVID-19 to the brain.

BBB is formed by several cellular components in the capillary-tissue interface such as pericytes and tight junctions between the endothelial cells. Its primary role is to protect the brain from neurotoxins and pathogens. Disruption of BBB will result in neuroinflammation and severe neural dysfunction and has been implicated in a number of neurological disorders. Mounting evidence suggested that BBB integrity is impaired due to neuroinflammation associated with SARS-CoV-2 infection in the acute phase [16]. Some studies further suggested that COVID-19 patients have chronically elevated levels of neuroinflammatory factors such as blood GFAP and MMP-9 [11]. However, to date, the chronic effect of severe COVID-19 on brain-specific BBB has not been reported. In this work, we evaluated the BBB integrity using non-invasive MRI and provided imaging-based evidence of neuroinflammation secondary to SARS-CoV-2 infection [17, 18]. It should be noted that this MRI technique does not require exogenous contrast agents and has been assessed across multiple vendors, making it easily scalable for larger studies [15].

Studies in other cognitive and neurological disorders have provided evidence that BBB permeability is related to cognitive function [19]. In Alzheimer’s disease, it has been shown that PS is inversed related to memory and composite cognitive scores and also associated with beta-amyloid and phosphorylated tau concentrations [20]. BBB permeability was also reported to be related to the E4 variant of apolipoprotein *E* (APOE4) genotype, which is a known risk factor for Alzheimer’s disease [21]. Therefore, it is plausible that disruption of BBB integrity in COVID-19 survivors will cause cognitive dysfunction. Elevated BBB permeability was also observed in sickle cell disease, which is characterized by microvascular damage due to abnormal red blood cells [22]. Thus, the BBB breakdown observed in COVID-19 patients could be due to a combined effect of microvascular and neuropathologic injuries.

It is reported that COVID-19 ICU survivors have an increased risk for long-term cognitive impairments [23]. BBB damage may allow neurotoxins to enter the brain and promote neurodegeneration [24]. Moreover, BBB dysfunction is likely to result in cerebral microbleeds, which were more prevalent in COVID-19 ICU survivors than general ward survivors [25, 26]. Given these plausible pathways, anti-inflammatory pharmacologic approaches and psychotropic medications, e.g., systemic corticosteroids [27], l-arginine [28], curcumin [29], vitamins [30], etc., may be considered to mitigate the long-term symptoms in COVID-19 survivors.

In addition to BBB permeability, this study investigated other brain anatomic and function parameters such as brain volume and CBF. We found that whole-brain volume and CBF were not significantly different between COVID-19 ICU survivors and controls, suggesting that BBB breakdown may be a more sensitive measure of brain dysfunction in neuro-long-COVID, in the absence of apparent anatomic or perfusion abnormalities.

The present study should be viewed as a pilot study given the small sample size of the COVID-19 ICU survivor group. Thus the findings reported in this work are meant to be used to generate hypotheses for further testing. Indeed, we are currently conducting a larger-scale study in long-COVID patients to verify the observations reported here. Since our control participants did not undergo ICU admissions, we cannot distinguish COVID-19 related effects from ICU stress and post-ICU syndrome [31]. Thus future work will include subjects admitted to ICU due to non-COVID diseases.

## Conclusions

Using a novel MRI technique, the present study provided preliminary evidence that COVID-19 survivors who have undergone severe infection in the acute phase manifested BBB abnormalities at the chronic stage, suggesting a long-lasting adverse effect of COVID-19 on the brain that may contribute to the cognitive symptoms in long-COVID.
